# KIAA1211 plays an oncogenic role in human non-small cell lung cancer

**DOI:** 10.7150/jca.35951

**Published:** 2019-10-22

**Authors:** Zhengcheng Liu, Hui Cao, Ye Shi, Rusong Yang

**Affiliations:** Department of Thoracic Surgery, Nanjing Chest Hospital, Nanjing, Jiangsu, 210000, China

**Keywords:** KIAA1211, NSCLC, Proliferation, Apoptosis

## Abstract

One of the main causes of cancer disease and death worldwide is lung cancer. Our study focused on the function of KIAA1211 in non-small cell lung cancer (NSCLC). According to the data about NSCLC patients that from the Cancer Genome Atlas (TCGA), we found that KIAA1211 in NSCLC (P=5.06E-06) was significantly higher than the adjacent normal. Lentivirus-mediated short hairpin RNA (shRNA) was used to knockdown BATF expression in the human A549 NSCLC cell line and assessed by RT-qPCR and Western blot. Cell proliferation was evaluated by MTT assay and Celigo imaging cytometry. Cell apoptosis were detected by Annexin V staining. The test results showed that KIAA1211-shRNA A549 and SPC-A-1 cells can inhibit cell proliferation, and the apoptosis rate of KIAA1211-shRNA group was significantly higher than that of the control group. Knockdown of KIAA1211 inhibited NSCLC progression in xenograft tumor model. In conclusion, KIAA1211 could regulate NSCLC cells proliferation and apoptosis *in vitro* and *in vivo*. KIAA1211 may serve as a potent target for the treatment of NSCLC.

## Introduction

Non-small cell lung cancer (NSCLC), accounting for approximately 80% of lung cancers, is one of the most malignant tumors worldwide with a low overall 5-year survival rate^1^, ^2^. Although several therapeutic agents for NSCLC have been developed, the 5-year survival rate of lung cancer is still 4-17% depending on stage and regional differences, which seriously influences on life quality of patients and brings a heavy burden to the family^3^. Much progress has been made in lung cancer screening, basic research, and personalised therapy in recent years^4^-^6^. Although great progress has been made in the study of the NSCLC, the molecular mechanisms remains largely unclear^7^. Thus, exploring the pathogenesis of NSCLC remains urgently to improve clinical treatment strategies.

KIAA1211 locates at human chromosome 4q12, and has only one transcript (NM_020722.1, 4914 bp)^8^. KIAA1211 encoded a 102-kDa nuclear protein and highly expressed in the brain cerebral cortex^9^-^11^. Furthermore, it is localized to the microtubules and the centrosomes and is subcellularly located in the nucleus^12^. In recent years, it has been found that the KIAA1211 gene is closely associated with the development and prognosis of various cancers^13^, ^14^. But the role of KIAA1211 in NSCLC has not been explored.

In the present study, the expression of KIAA1211 for NSCLC inTCGA database was investigated. In order to study the function and effect of KIAA1211 in NSCLC, we have carried out a series of experiments. Besides, the influence of KIAA1211 on tumor formation *in vivo* was testified in mice. These results provide important information to explain the pathogenesis of NSCLC, and also reveal that KIAA1211 may be a new potential target for the treatment of NSCLC.

## Materials and Methods

### The expression level of KIAA1211 in TCGA database

TCGA is a publicly funded project and has produced data at the RNA level for various cancers, including NSCLC. The mRNA expression profiling data of NSCLC and matched adjacent mucosa in TCGA was downloaded. We calculated the differences in KIAA1211 expression between adjacent non-tumor tissues and NSCLC, and used histogram to describe these differences.

### Cell lines and culture conditions

NSCLC cell lines A549 and SPC-A-1 were were obtained from the Shanghai Cell Bank (Shanghai, China). All cells were cultured in Roswell Park Memorial Institute 1640 (Thermo Fisher Scientific, Waltham, MA, USA) supplemented with 10% fetal bovine serum (FBS; Thermo Fisher Scientific), 100 IU/mL penicillin (Sigma, St Louis, MO, USA), and 100 µg/mL streptomycin (Sigma).

### Real-time Quantitative PCR (RT-PCR) for Endogenous KIAA1211 Expression Detection in NSCLC Cell Lines

cDNA was synthesised from total RNA (500 ng) in a 10-μl reaction volume using a PrimeScript RT-PCR Kit (TaKaRa, USA). Then, qPCR was performed using the power SYBR Green master mix (Thermo Fisher Scientific). The relative expression levels were calculated using 2^-ΔΔCt^ method following normalization against GAPDH for KIAA1211. The primers were used as follows:

- KIAA1211-F, 5'- CCTCCCGTCTTTCCTTGTCCC -3';
- KIAA1211-R, 5'- CTGGCTTTGCTATCCCGTTTG -3';
- GAPDH-F, 5'- TGACTTCAACAGCGACACCCA -3';
- GAPDH-R, 5'- CACCCTGTTGCTGTAGCCAAA -3'.

### Lentiviral shRNA vector construction

The KIAA1211 (Gene ID: 57482) mRNA sequences were obtained from the NCBI database (http://www.ncbi.nlm.nih.gov/pubmed). The lentiviral vector pGVX115-GFP by Genechem (Shanghai, China) contained a human H1 polymerase-III promoter for shRNA expression. The pGVX115-GFP was used as negative control and was named shCtrl.

The lentiviral vector pGVX115-GFP by Genechem (Shanghai, China) contained a human H1 polymerase-III promoter for shRNA expression. Each shRNA insert was designed as a synthetic duplex with overhanging ends identical to those created by BamHI restriction enzyme digestion at the 5' end and HindIII restriction enzyme digestion at the 3' end. There was a nine-nucleotide ring sequence (TTC AAG AGA) between the strand and antisense strands. Following digestion by the BamHI/HindIII restriction endonucleases, the two sequences were inserted into the pGVX115 plasmid by ligating the same restriction enzyme-digested shRNA fragment to the vector.

### Cell Proliferation and Colony Formation Assays

In our study, cell growth status was analyzed by two methods, the Celigo imaging cytometry system and MTT assays. The fluorescence intensity of cells was scanned and the number of cells was automatically calculated by Celigo imaging cytometry system (Nexcelom, Lawrence, MA, USA). Cell viability was measured with a Cell Viability Kit (MTT, Roche, Indianapolis, IN, USA) according to the manufacturer's instructions. The absorption of the solution was measured at 570 nm at various time points.

Colony formation assays were performed to assess the ability of a single cell to grow into a colony. Cells were plated at a low density (500-1000 cells/well) onto 6 well plates and observed for 2 weeks. Colonies were fixed in 4% paraformaldehyde, stained with 0.5% crystal violet staining solution and washed with PBS.

### Apoptosis Assay

Cells were incubated in 6-well plates for 24 hours and treated with shBATF and shCtrl for 48 hours. The cells were washed, and then the Annexin V-APC Apoptosis Detection kit was used according to the manufacturer's guidelines to assess apoptosis. After incubation in the dark for 15 min, cells were analyzed by FACS Calibur flow cytometer (Becton Dickinson, USA). Each experiment was performed in triplicate.

### Cell proliferation assay and tumor formation assay in xenograft tumor mouse models

Five-week BALB/c nude mice were purchased from Experimental Animal Center of Soochow University, and all animal protocols were approved by the Institutional Animal Care and Treatment Committee of Soochow University. The BALB/c nude mice were randomly divided into two groups (n = 6/group). One group of mice was inoculated subcutaneously with PANC-1/Vector cells (2 × 106) in the left dorsal flank and with PANC-1/ GPR87 cells (2 × 106) in the right dorsal flank per mouse. Another group was inoculated subcutaneously with PANC-1/RNAi-vector cells (2 × 106) in the left dorsal flank and with PANC-1/GPR87-RNAi cells (2 × 106) in the right dorsal flank. Tumors were examined twice weekly; length and width measurements were obtained with calipers and tumor volumes were calculated using the equation (L ×W2)/2.

### Statistical Analysis

Statistical analysis was performed using GraphPad Prism 5 software. Statistical differences were analyzed and calculated by a one-way analysis of variance (ANOVA) or unpaired two-tailed t-test, and statistical significance was obtained when *P* was < 0.05. * Indicated *P* < 0.05; ** indicated *P* < 0.01 and *** indicated *P* < 0.001.

## Results

### The expression levels of KIAA1211 in TCGA dataset

**Figure 1:**
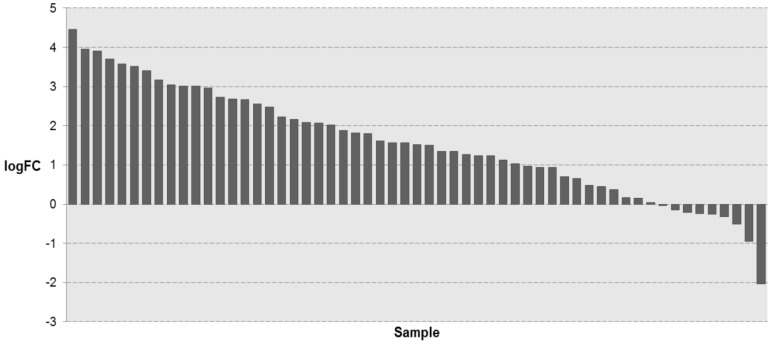
The expression level of KIAA1211 in lung cancer was detected in TCGA database. The logFC of the expression level of KIAA1211 in lung cancer compared to adjacent normal tissues based on TCGA dataset.

To investigate whether KIAA1211 expression was altered in NSCLC tissues, we retrieved the mRNA expression profiling of NSCLC tissues and adjacent normal tissues from TCGA database. As shown in **Figure 1**, the mRNA expression of KIAA1211 in NSCLC tissues was significantly higher than that in adjacent non-tumorous tissues (P < 0.001).

### Knockdown Efficiency of KIAA1211 by shRNA Lentivirus Infection in NSCLC cell lines

**Figure 2:**
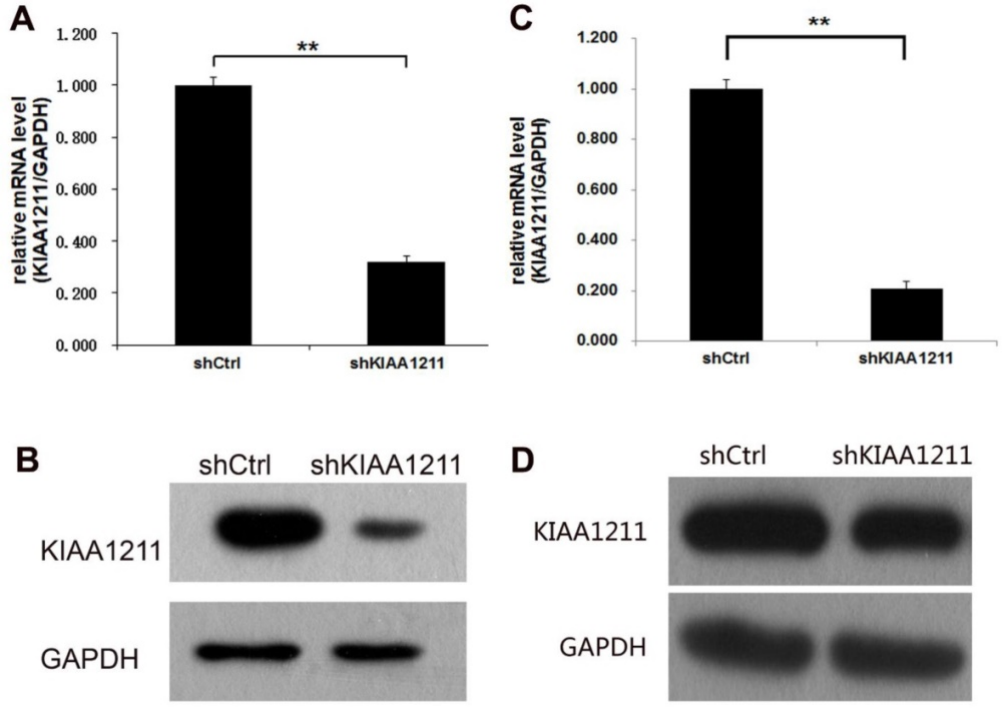
The expression of KIAA1211 both in A549 and SPC-A-1 cell lines was detected through RT-PCR and Western Blot analysis after shKIAA1211/shCtrl infection. (A) the mRNA expression level of KIAA1211 in A549 cell lines was detected through RT-PCR analysis; (B) the protein expression level of KIAA1211 in A549 cell lines was detected through Western Blot analysis. (A) the mRNA expression level of KIAA1211 in SPC-A-1 cell lines was detected through RT-PCR analysis; (B) the protein expression level of KIAA1211 in SPC-A-1 cell lines was detected through Western Blot analysis.

To investigate the role of KIAA1211 in A549 and SPC-A-1 cell lines, lentivirus-sh KIAA1211 (shKIAA1211) and lentivirus-shCtrl (shCtrl) were used to infect A549 and SPC-A-1 cell lines. Transfection efficiency was evaluated using RT-PCR assay and the results showed that KIAA1211 mRNA level was reduced by 80% in shKIAA1211 group (**Figure 2A**). Compared to cells infected with shCtrl group, KIAA1211 protein expression was downregulated by shKIAA1211 lentivirus in A549 and SPC-A-1 cell lines** (Figure 2B)**.

### Knockdown of KIAA1211 Inhibited Cell Proliferation and Colony Formation of NSCLC cell lines

**Figure 3:**
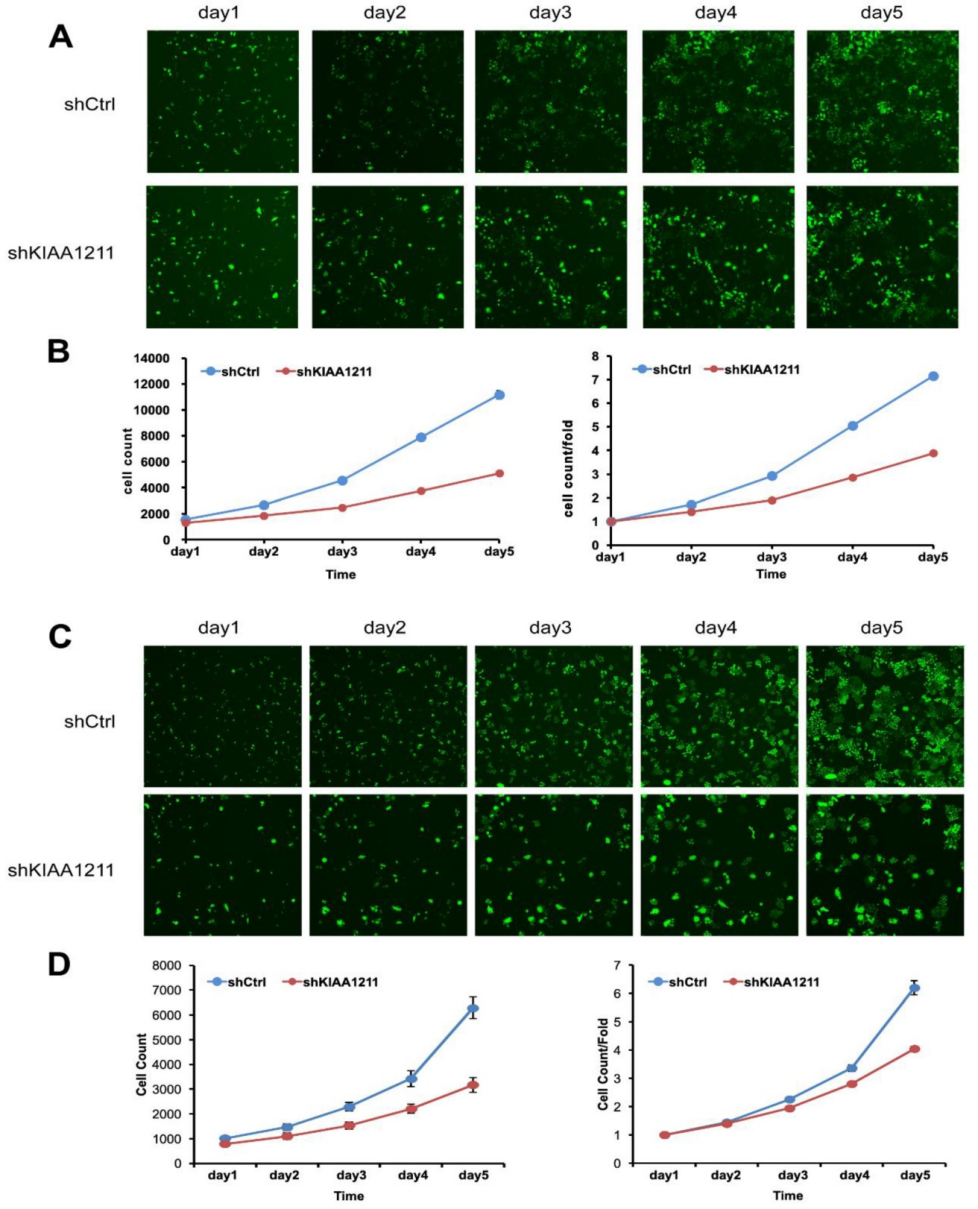
Cell growth of A549 and SPC-A-1 after shKIAA1211/shCtl infection was detected by Celigo calculation. (A) Cell growth of A549 after shKIAA1211/shCtrl infection from day 1 to day 5; (B) cell growth curves of A549 was depicted according to Celigo calculation; (C) cell growth of SPC-A-1 after shKIAA1211 /shCtrl infection from day 1 to day 5; (D) cell growth curves of SPC-A-1 was depicted according to Celigo calculation.

**Figure 4:**
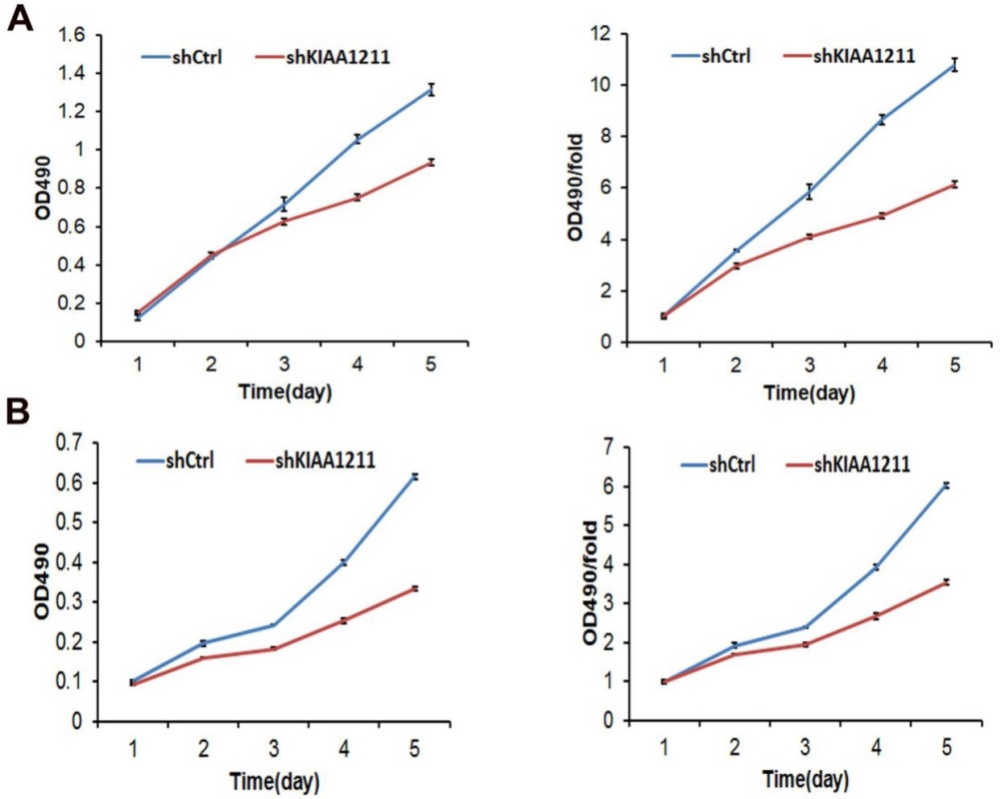
Cell growth of A549 and SPC-A-1 cell lines after shKIAA1211 and shCtrl infection were examined through MTT assay. (A) Cell growth of A549 after shKIAA1211 /shCtrl infection from day 1 to day 5; (B) cell growth of SPC-A-1 after shKIAA1211 /shCtrl infection from day 1 to day 5.

**Figure 5:**
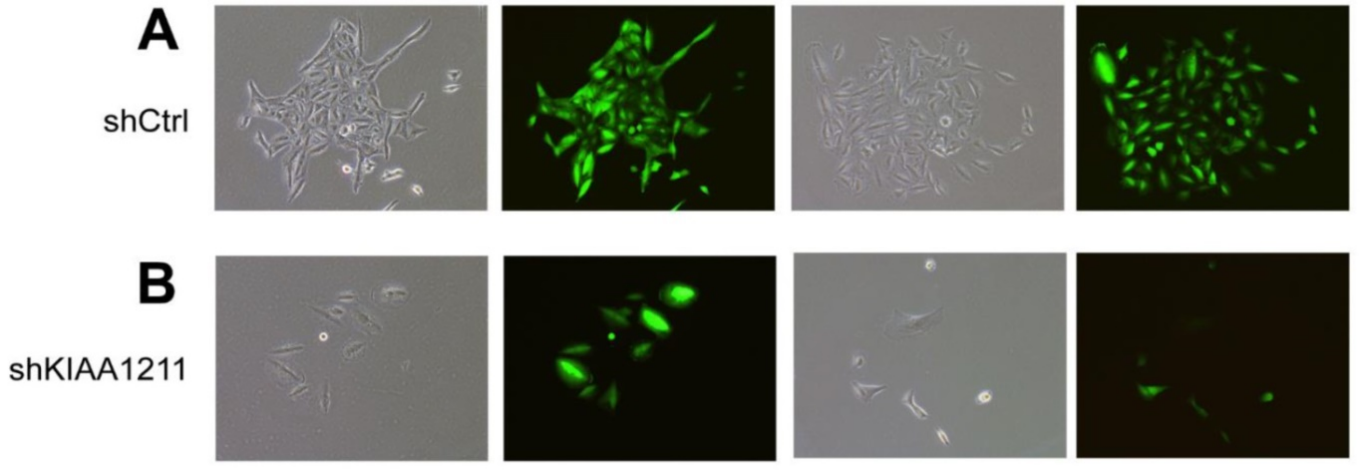
Colony formation efficiency of A549 and SPC-A-1 cells were observed after shKIAA1211/shCtrl infection for 11 days. (A) Cell colony of A549 cells in shKIAA1211 and shCtrl group; (B) Cell colony of SPC-A-1 cells in shKIAA1211 and shCtrl group.

We explored the role of shKIAA1211 in the proliferation and colony formation abilities of A549 and SPC-A1 cells after infected with lentivirus with shKIAA1211 and control group in a 5-day study. Celigo Imaging Cytometry System (**Figure 3**) and MTT assay (**Figure 4**) revealed that KIAA1211 knockdown obviously decreased cell proliferation of A549 cells at 48 h-96 h. Moreover, cell colony assay showed that KIAA1211 knockdown significantly decreased the number of cell colony of A549 and SPC-A1 cells** (Figure 5)**.

### Knockdown of KIAA1211 induced the cell apoptosis in A549 and SPC-A-1 Cells

As seen in Figure 5, the percentage of apoptosis increased to 8.02%±0.48 in A549 and 12.01±0.21% in SPC-A-1 cells after shKIAA1211 lentivirus infection, which was significantly higher than the control (A549: 5.31±0.04%; SPC-A-1: 3.96±0.12%), suggesting that shKIAA1211 promoted NSCLC cell apoptosis.

### Knockdown of KIAA1211 inhibited NSCLC progression *in vivo*

**Figure 7:**
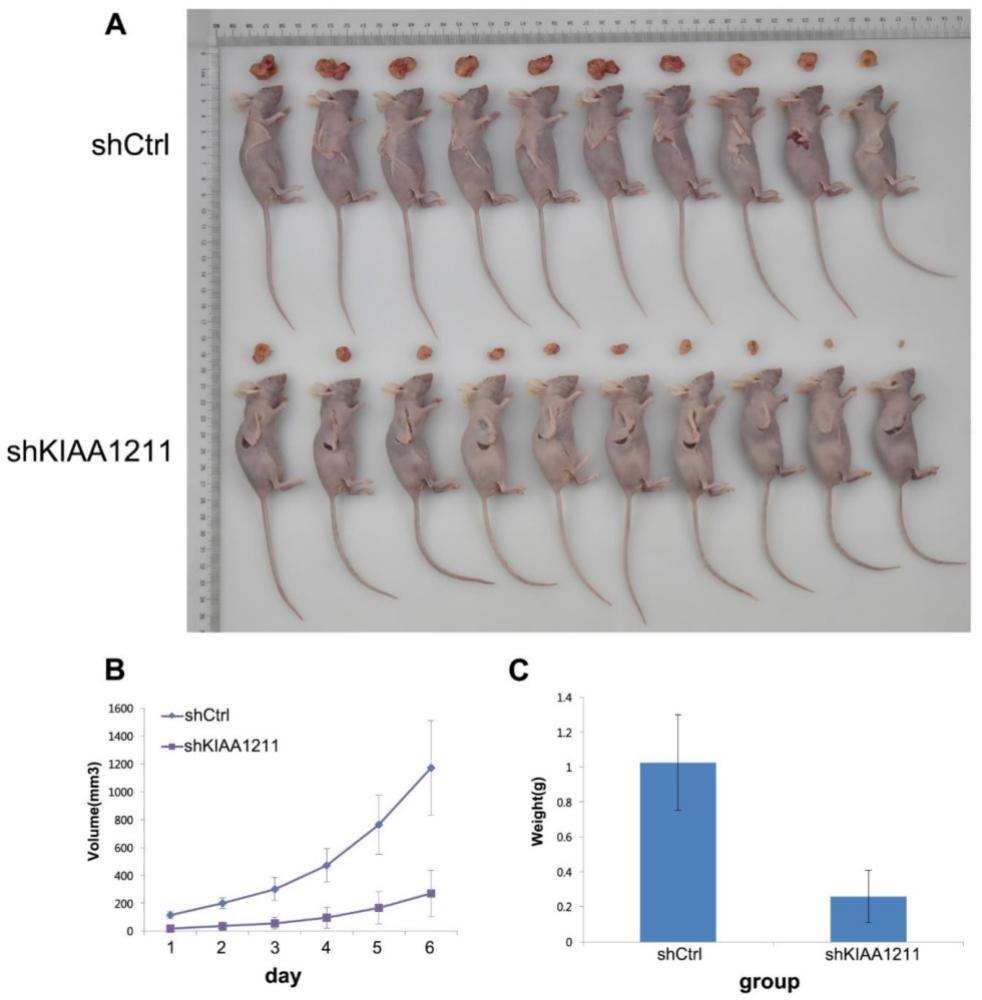
Effect of KIAA1211 on tumor progression *in vivo*. (A) Xenograft tumor mice and tumor were photographed. (B) Tumor volumes were measured on the indicated days. (C) Mean tumor weights in shKIAA1211 and shCtrl group.

To examine the biological effects of KIAA1211 on NSCLC progression, a xenograft tumor model was used. As shown in **Figure 7**, tumors formed by KIAA1211-silenced A549 cells were smaller and had lower tumor weights than the shCtrl tumors.

## Discussion

In spite of improvement in adjuvant chemoradiotherapy and surgical techniques have been made in NSCLC treatment, the 5-year survival rate of NSCLC increase, but it is still below 17%. The development of NSCLC involves both environmental and genetic changes^15^, and the activation of oncogenes influence the proliferation, adhesion, motility, invasiveness of cacer cells and reconstruct the contact of tumor cells and surrounding extracellular matrix, promoting EMT process^16^, ^17^. Therefore, the identification of novel biomarkers is critical for the improvement of clinical outcomes for NSCLC patients.

KIAA1211 gene is an important member of the KIAA gene family that in the HUGE database. The gene expression of KIAA1211 is tightly controlled by both genetic and epigenetic regulatory mechanisms. Estrogen-related receptor alpha (ERRα) is a notable transcription factor due to low expression levels of KIAA1211 when estrogen receptors are knocked down^18^. KIAA1211 is thought to be a cancer-promoting gene, overexpressed in many types of tumors, especially colorectal cancer, stomach cancer, breast cancer, etc^19^, ^20^. Recent studies reported that KIAA1211 was associated with certain mental disorders and various cancers^9^. Spurrell and colleagues have identified KIAA1211 for inherited breast cancer by exome sequencing^21^.

In this report, we provide several lines of evidence demonstrating that KIAA1211 plays an oncogenic role in human NSCLC. First, we found that KIAA1211 in NSCLC (*P*=5.06E-06) was significantly higher than the adjacent normal according to the data about NSCLC patients that from the Cancer Genome Atlas (TCGA). Second, KIAA1211-shRNA A549 and SPC-A-1 cells can inhibit cell proliferation, and the apoptosis rate of KIAA1211-shRNA group was significantly higher than that of the control group. Third, knockdown of KIAA1211 inhibited NSCLC progression in xenograft tumor model. Based on the above research results, KIAA1211 could regulate NSCLC cells proliferation and apoptosis *in vitro* and *in vivo*. Therefore, our work has established the basis for further investigation of KIAA1211 and its oncogenic roles in NSCLC.

In summary, our results showed that KIAA1211 is overexpressed in NSCLC tissues in TCGA database. Knockdown of KIAA1211 can inhibit cell proliferation and cell colony formation capacity, and promote cell apoptosis in NSCLC cell lines. Knockdown of KIAA1211 also inhibited NSCLC progression *in vivo*. Despite the precise molecular mechanism surrounding malignant behavior attenuation needs further clarification, but this discovery may provide new insights for the tumorigenesis and tumor progression in NSCLC. Therefore, KIAA1211 may serve as a potent target for the treatment of NSCLC.

## Figures and Tables

**Figure 6:**
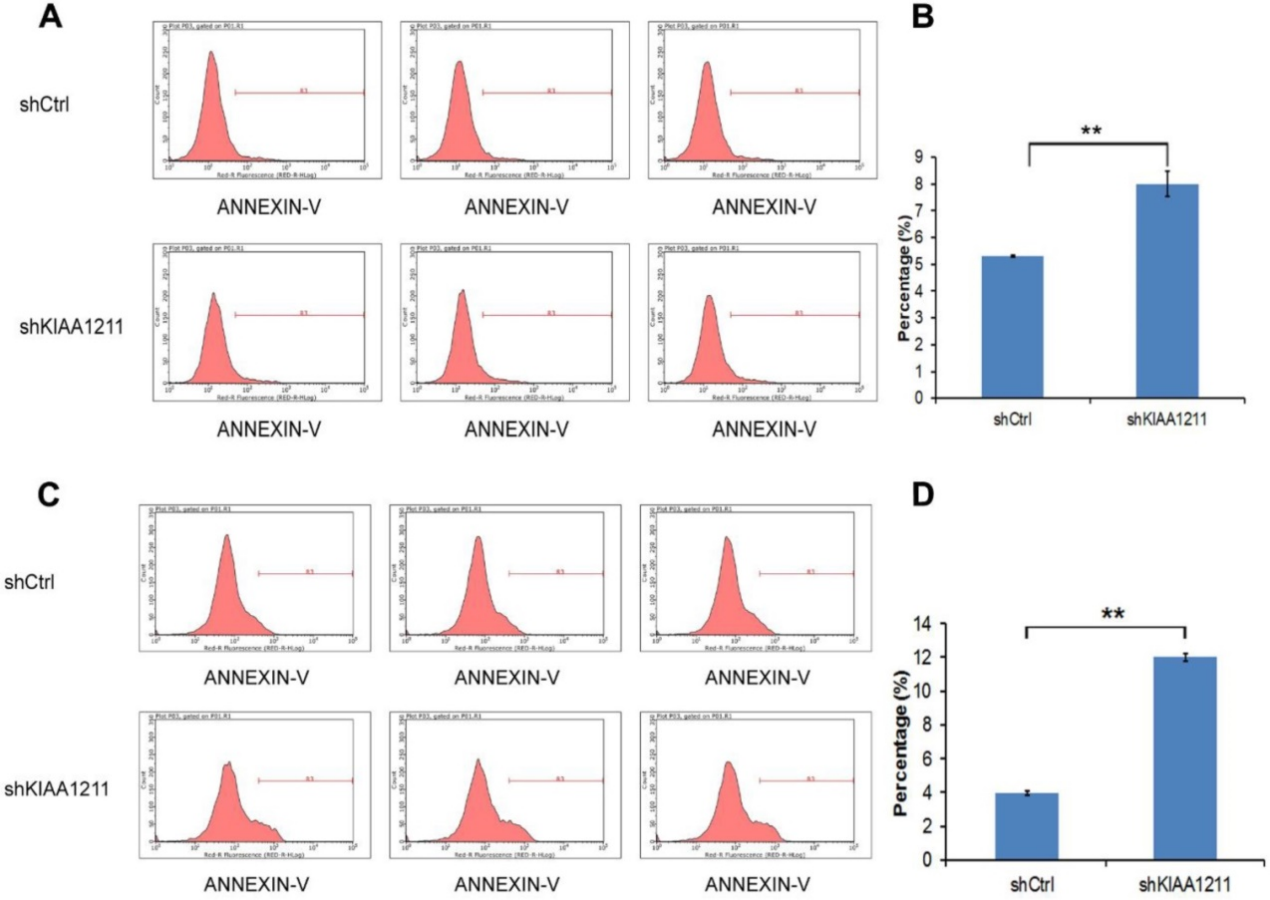
Cell apoptosis of A549 and SPC-A-1 cell lines after shKIAA1211 and shCtrl infection were examined through flow cytometry. (A) Cell apoptosis analysis in A549 cells with shKIAA1211 or shCtl infection; (B) the percentage of cell apoptosis in A549 cells; (C) cell apoptosis analysis in SPC-A-1 cells with shKIAA1211 or shCtl infection; (D) the percentage of cell apoptosis in SPC-A-1 cells.
